# How Does Entrepreneurial Team Relational Governance Promote Social Start-Ups’ Organizational Resilience?

**DOI:** 10.3390/ijerph19116677

**Published:** 2022-05-30

**Authors:** Yingping Mai, Yenchun Jim Wu, Yu-Min Wang

**Affiliations:** 1 Business School, Huaqiao University, Quanzhou 362021, China; myp@stu.hqu.edu.cn; 2 Graduate Institute of Global Business and Strategy, National Taiwan Normal University, Taipei City 10645, Taiwan; 3 College of Humanities and Arts, National Taipei University of Education, Taipei City 10671, Taiwan; 4 School of Innovation, Entrepreneurship and Creation, Minjiang University, Fuzhou 350108, China

**Keywords:** entrepreneurial team, social entrepreneurial team relational governance, organizational resilience, team learning, social entrepreneurship, social start-ups

## Abstract

Why are some social entrepreneurial teams able to adapt to challenges and leverage the opportunities that are generated from a crisis, and why can some start-ups achieve sustained growth yet others do not? From the perspective of relational governance, this study unpacked the mechanism of how entrepreneurial teams promote social start-ups’ abilities to deal with crises and the mediating role of team learning through a survey of 396 social entrepreneurial team members. The results showed four key findings. (1) Trust among entrepreneurial team members has a positive effect on organizational resilience, whereas shared vision and communication-cooperation do not. (2) All the dimensions of relational governance positively promote team learning, and team learning is positively associated with organizational resilience. (3) Team learning mediates the effect of entrepreneurial team relational governance on organizational resilience; specifically, team learning plays a complete intermediary effect on shared vision and communication-cooperation to organizational resilience, whereas it plays a partial intermediary effect on trust in organizational resilience. (4) Team learning is the key factor to organizational resilience, whereas communication-cooperation promotes team learning the most. Practically, to strengthen social start-ups’ organizational resilience, entrepreneurial team members must first improve their understanding of environmental adaptability and then engage in productive and creative dialogues to manage issues, improve team members’ capability in information integration, as well as agree upon the action and activities that should be performed.

## 1. Introduction

Entrepreneurial team members are the owners and top managers who play critical roles in the survival and development of new social enterprises, especially in a turbulent environment [1,2]. In previous studies, which are mainly based on upper echelons theory, scholars examined the relationship between team composition and firm performance. Their results showed that the cognition, values, and demography of top management teams significantly influenced their abilities of environmental condition interpretation and reflection on firm behavior [3,4]. However, entrepreneurial team ability is not a simple superposition of each team member’s ability. The desired team cooperation and giving full play to the team advantage of 1 + 1 > 2 is difficult to achieve if team institution is lacking, even if team composition is reasonable. Therefore, scholars argued that a team composition perspective cannot open the black box between the entrepreneurial team and the growth of the firm, and exploring the mechanism of the entrepreneurial team on the sustainable growth of start-ups is necessary from the perspective of institution [5,6]. The same is true for the field of social entrepreneurship. From the perspective of entrepreneurial process, Mair and Marti [7] defined social entrepreneurship as the innovative use of resource combinations to pursue opportunities aiming at the creation of organizations and/or practices that yield and sustain social benefits, and Austin et al. [8] suggested that these kinds of innovation and social value creation activities can be found within or across non-profit organizations, enterprise, or government departments, no matter where social entrepreneurship comes from, the same destination they will go to is regarding social value creation as the necessary condition of the entrepreneurship activities [9]. In other words, formal contracts that aim to encourage economic creation might fail to stimulate the continuous entrepreneurial engagement of the team members as social entrepreneurs do not make money for themselves, and entrepreneurial team relational governance aims to prepare team members to share vision, cooperation-communication, and trust is more advanced to encourage team members to exert all their strength to realize their goals in social benefits. Furthermore, unlike mature enterprises that already exist with widely social networks, sufficient resource based, and fixed operating routines to respond to the fast changing environment, social start-ups can only improve their abilities to respond to unexpected issues through trial and error. This makes entrepreneurial team governance is better than formal contracts because the former enables the team members to evolve their collaboration along with the dynamic environment while the latter might lead to organizational rigidity from the limitation of contract incompleteness.

Entrepreneurial team learning is considered a crucial way for start-ups to respond to a dynamic environment and achieve sustainable growth. The more unfavorable the environment is, the more entrepreneurs and top managers need to adapt to new challenges and leverage new opportunities in the environment through learning [10,11]. Similarly, social entrepreneurs and top managers can improve their human capital and capacities, so as to promote the ability of social enterprise, and finally enable the social enterprise to cope with the impact of unexpected changes and achieve sustainable growth. In the context of entrepreneurship, Chen et al. [12] believed that learning is a socialized process in which entrepreneurial team members solve problems and accumulate knowledge together on the basis of shared rules and procedures. Therefore, in this study, team learning is introduced as an intermediary variable to help deepen the understanding of the role of how entrepreneurial team relational governance promotes social start-ups’ organizational resilience. In summary, this study aims to explore two questions: (1) whether and how does entrepreneurial team relational governance improve the organizational resilience of social start-ups? (2) What mediating effect does team learning play between them?

To answer the questions, the rest of this study is organized into five sections. Specifically, Section 2 introduces the background of the related variables and then proposes the hypotheses and research framework. Section 3 presents the methodology, including the scales, the method of data collection, and the demography of the participants. Section 4 shows the results of the data analysis, including the reliability test, the results of the hypothesis test, and explores further insights into the results through Importance–Performance Map Analysis (IPMA). Section 5 discusses the results and Section 6 puts forward the contribution, limitations, and future research directions.

The results showed that trust among entrepreneurial team members promotes social start-ups’ organizational resilience directly whereas shared vision and communication-cooperation do not. Team learning plays an important intermediary role between entrepreneurial team relational governance and organizational resilience. This study makes two main contributions. (1) Theoretically, it strengthens the application of institutional theory in the field of social entrepreneurship and explores and verifies the roles of stimulating entrepreneurial team members to produce “chemical reactions” to promote organizational resilience. (2) Practically, the findings offer clear guidance for social start-ups to improve their organizational resilience and thus expand their social impact.

## 2. Theoretical Background and Hypotheses

### 2.1. Theoretical Background

Entrepreneurial team heterogeneity is a double-edged sword. The diverse backgrounds of entrepreneurial team members provide greater knowledge and value, which may help social enterprises achieve their dual goals. However, if coordination is lacking, then the heterogeneity of a social entrepreneurial team will also lead to inconsistent or antagonistic actions within the team [13,14]. This scenario may limit the team’s ability to give full play to their advantages and even lead to social entrepreneurial failure. On the basis of institutional theory, this study suggests that team institution is the foundation of entrepreneurial team cooperation. According to institutional theory, an institution is a rule that guides participants on what can or cannot be done and how to do it, which limits participants’ opportunistic behavior as well as makes interpersonal communication transparent and includes formal institution and informal institution [5]. Based on this, entrepreneurial team institution includes contractual governance and relational governance. Furthermore, as suggested by Peng [15], informal institution has its unique advantages in a dynamic environment, and the social value creation orientation of social start-ups. This study focuses on entrepreneurial team relational governance and constructs its theoretical relationship with organizational resilience.

On the basis of the studies of relational governance in the field of corporate governance, alliance, and family business governance, Zhu et al. [16] suggested that entrepreneurial team relational governance is a team norm to format good team interactions. It can be divided into three dimensions: shared vision, communication-cooperation, and trust. First, unlike general top managers who are only taking responsibility for the firm, entrepreneurial team members usually share a vision and work together to realize the same goal [17]. Second, adequate communication-cooperation is needed for entrepreneurial team members to integrate their heterogeneous knowledge well to achieve an outcome of 1 + 1 > 2 [18]. Third, mutual trust among team members makes the team flexible and adaptive [19]. Thus, the interdependence among entrepreneurial team members determines that they must adopt relational governance to form a shared vision, improve their communication-cooperation, and fully trust one another during the entrepreneurial process.

Team learning is believed to be the foundation of organizational learning [20]. It plays a great influence on preparing a team to be effective in achieving their common goals [21], and finally enables the firm to adapt to unexpected breaks. An unexpected break is often without existing coping strategies or experience, which requires entrepreneurs and top managers to search for new solutions, thus pushing them to improve their ability to tackle the negative situation by learning. In other words, a dynamic environment pushes firms to improve their organizational resilience, and entrepreneurs and top managers are required to develop effective coping strategies through continued learning to ensure firms can run smoothly. However, due to the limited rationality of humans, the knowledge and ability of a single social entrepreneur may not be powerful enough to overcome the dynamic environment [22], and mutual support among entrepreneurial team members is required to create greater knowledge and ability for the firm [23]. Thus, a team institution is needed to improve the atmosphere of team learning to encourage team members to share and exchange their knowledge with others.

Meyer [24] introduced organizational resilience and suggested that organizational resilience stems from a firms’ response to environmental turbulence, which helps the firm maintain or rebuild its structure and function through dynamic coping strategies. Since then, scholars have expanded the concept from different perspectives. Sincorá et al. [25] suggested that organizational resilience can be seen as a form of dynamic capability, which enables organizations to absorb pressure, perceive in a timely manner, correct adverse trends in the early stage, and actively respond to unexpected situations to enable firms better avoid risks in the future, thus improving their ability to deal with negative events or unexpected breaks [26,27]. Organizations with high resilience are believed to survive and recover sooner in adverse circumstances [28,29] because they can adjust and re-establish strategies and business models according to environmental changes quickly [30]. Thus, social start-ups need to cultivate their organizational resilience as the environment is always changing, such as rapid technological changes, political transformation, and sudden public crises.

### 2.2. Entrepreneurial Team Relational Governance and Social Start-Ups’ Organizational Resilience

As the leader and decision-maker, the entrepreneurial team plays a critical role in cultivating organizational resilience [31], including building an organizational environment with mutual trust and support, improving the quality of decision-making, and even determining the survival and development of the organization [32,33]. Scholars believe that the heterogeneity of the top management team offers multiple abilities to perceive opportunities and crises and helps the team carry out suitable strategies through comprehensive analyses [34]. Other scholars others have argued that the heterogeneity in beliefs, attitudes, and values may not be conducive to team building [35]. Similarly, in the context of social entrepreneurship, diverse knowledge and ability are good for a social enterprise in realizing its dual mission. However, they may also cause team conflict or entrepreneurial failure without correct governance. Therefore, a governance structure that supports collective action and links decentralized fields from different institutional frameworks is needed to help generate organizational resilience [36]. In practice, entrepreneurial teams are always required to respond to a sudden attack quickly under insufficient communication conditions. Team members must trust one another and implement the coping strategy for crises unconditionally and quickly. Thus, entrepreneurial team relational governance is positively associated with the ability of social start-ups to deal with crises. First, as social entrepreneurship needs to balance its social and economic missions, a shared vision is a lighthouse that guides an entrepreneurial team to choose the correct strategies and avoid mission drift. Second, communication-cooperation not only enables team members to fully communicate, it also helps them alleviate the negative impact of team conflicts and enhances the willingness of team members to exert more effort to achieve their organizational goals. Finally, information and orders may feed back and transmit rapidly if the team members trust one another [37]. In summary, entrepreneurial team relational governance not only encourages benign interactions among team members and makes their behavior predictable but also helps make the organizational structure flexible, so as to promote the ability of social start-ups to deal with unexpected issues.

**Hypothesis** **1a** **(H1a).** *Shared vision of entrepreneurial team members is positively associated with the social start-ups’ organizational resilience.*

**Hypothesis** **1b** **(H1b).** *Communication-cooperation of entrepreneurial team members is positively associated with the social start-ups’ organizational resilience.*

**Hypothesis** **1c** **(H1c).** *Trust among entrepreneurial team members is positively associated with the- social start-ups’ organizational resilience.*

### 2.3. Entrepreneurial Team Relational Governance and Team Learning

Organizational learning is the collective learning of top managers’ socialized interaction because they are the key agents of an organization [38], and their cognition and values reflect the strategies of the organization. In the context of entrepreneurship, Chen, Fu, Zhang, and Yu [12] believed that learning is the socialization of entrepreneurial team members, such as solving a problem and accumulating knowledge on the basis of shared rules and procedures, to integrate necessary information and knowledge to promote start-ups’ growth and realize their mission. Nonaka, o Nonaka, Ikujiro, and Takeuchi [18] believed that constructing a “field” is an effective way to encourage members to communicate and share knowledge, which will improve their cognition in opportunity and crisis identification. Thus, a team institution is necessary to promote effective learning, and entrepreneurial team relational governance positively incentivizes team learning. First, shared vision refers to the common and heartfelt will of all the entrepreneurial team members for the mission of the firm [17]. A shared vision may make the entrepreneurial team member know the future development direction of the social start-up clearly, and thus enables the team to share and absorb knowledge pertinently. Moreover, limited by the underdeveloped external network of social start-ups and the human capital of entrepreneurial team, members may not be fully tapped and internal learning will be more practical to the social entrepreneurial team, which means the entrepreneurial team may learn from their colleagues instead of sourcing knowledge outside. According to Nonaka, o Nonaka, Ikujiro, and Takeuchi [18], communication-cooperation is a necessary condition for team learning because it is almost the only way for tacit knowledge sharing, which means only when the team members communicate smoothly and cooperate frequently can they share and acquire knowledge effectively. Finally, trust is widely accepted as the most critical premise to promote team learning. However, vigilance among entrepreneurial team members is inevitable [39], especially among those with proprietary skills or knowledge know-how. They might worry that their knowledge will be stolen by other members; hence, they hide their knowledge. Thus, cultivating trust among entrepreneurial team members is necessary to dispel their concerns and encourage them to share knowledge.

**Hypothesis** **2a** **(H2a).** *Shared vision of entrepreneurial team members is positively associated with team learning.*

**Hypothesis** **2b** **(H2b).** *Communication-cooperation of entrepreneurial team members is positively associated with team learning.*

**Hypothesis** **2c** **(H2c).** *Trust among entrepreneurial team members is positively associated with team learning.*

### 2.4. Team Learning and Social Start-Ups’ Organizational Resilience

The ability of the top management team to perceive threats and identify opportunities is crucial to organizational resilience [40,41]. Such an ability consequently determines their abilities in decision-making and identifying opportunities that are brought about by crises [42], especially in a dynamic environment. Entrepreneurial team learning is one of the most important factors to achieve successful social entrepreneurship. First, a social entrepreneurial team is likely to have a team conflict because social entrepreneurship pursues dual goals, namely, social mission and economic benefits. Without learning, social entrepreneurial team members may not understand the logic that is followed by each other as some members might pursue social value creation whereas others might prefer economic profit output. These differences may cause an imbalance in the organizational mission and the personal needs of team members, ultimately resulting in mission drift. Through team learning, social entrepreneurial team members not only can fully share information and knowledge to improve their team knowledge level, but also can eliminate the deficiencies in their work, which may improve the relationship among the team members and finally improve the team’s decision-making quality [43]. Second, social entrepreneurship prefers to settle social needs in innovative ways, which requires a high ability to break through or reshape the existing system. In such a context, only team members that update their knowledge in both business and society in a timely manner can innovate solutions that could fulfill unmet social needs [44]. Hence, they are able to grasp the opportunities that are brought about by unfavorable conditions better [11,20]. To sum up, through team learning, the team may improve their cognition, which helps them correctly interpret a crisis and thus generate suitable strategies to enable the social enterprises to adapt to the dynamic environment and achieve sustainable growth.

**Hypothesis** **3** **(H3).** *Entrepreneurial team learning is positively associated with the social start-ups’ organizational resilience.*

### 2.5. The Mediating Effects of Team Learning

Entrepreneurial team relational governance does not only encourage team members to share their knowledge but also enables them to accept and absorb new knowledge from the other team members, which may accelerate the knowledge integration and create new knowledge and thus generate collective wisdom. The higher the team’s learning level, the higher the team’s knowledge level, and the better the social entrepreneurial team can identify unmet social needs that are caused by a crisis and take opportunities to expand their social impact. First, sufficient knowledge is needed to achieve the mission of the social enterprise, which may lead to more discussion among team members [45], so that the team will better understand their organization and adjust their strategies to match the fast-changing environment. Second, the more communication-cooperation among the social entrepreneurial team members, the more knowledge will be created [46]. This situation stimulates the team to generate a variety of decision-making skills and prepares them for quick emotional adjustment when routine procedures are disrupted, thus reducing the vulnerability of the social enterprise. Third, although conflicts in goals and values are inevitable within a social entrepreneurial team, as long as the social entrepreneurial team members trust one another, they will believe that all decisions that are made by the team are for the good of the social enterprise. They can formulate response strategies and take actions to deal with the crisis unconditionally and quickly. Thus, relational governance improves the learning atmosphere of entrepreneurial teams and hastens the knowledge transfer and absorption within the team, to improve their knowledge sharing and flexibility [47].

**Hypothesis** **4a** **(H4a).** *Entrepreneurial team learning mediates the relationship between their shared vision and the social start-ups’ organizational resilience.*

**Hypothesis** **4b** **(H4b).** *Entrepreneurial team learning mediates the relationship between their communication-cooperation and the social start-ups’ organizational resilience.*

**Hypothesis** **4c** **(H4c).** *Entrepreneurial team learning mediates the relationship between their trust and social start-ups’ organizational resilience.*

**Figure 1:**
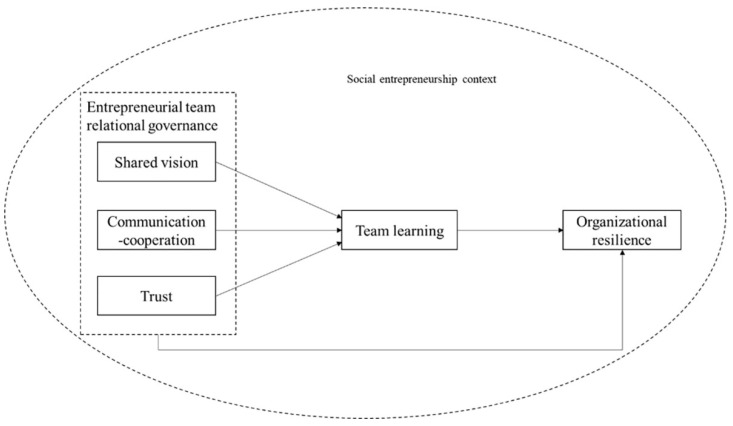
Theoretical Framework Design.

Figure 1 shows the theoretical framework of this study based on the hypotheses.

## 3. Materials and Methods

### 3.1. Questionnaire Design

The first draft of the questionnaire was formed by a multidisciplinary team composed of social entrepreneurs and senior scholars in the field of entrepreneurship. Next, the official questionnaire was validated by the team in two steps. (1) To make the questionnaire readable and understandable, the questionnaire was sent to social entrepreneurial team members, including founders and top managers to seek suggestions for further improvement. (2) A small-scale pre-survey was conducted to test the reliability and stability of the questionnaire. The final version of the questionnaire included two parts. The first part collected demographic characteristics of the participants and their company, including gender, age, education, position, company age, and company size; and the second part was a measure of the theoretical structure of the model, including the scales of shared vision, communication-cooperation, trust, team learning, and organizational resilience. The five constructs of the model were based on 35 questions, as shown in Appendix A, Table A1. The participants gave their answers using a seven-point Likert scale, where 1 indicates “totally disagree” and 7 indicates “totally agree”.

According to the literature review, social entrepreneurial team relational governance includes three dimensions, namely, shared vision, communication-cooperation, and trust. The relevant scales that have been proven to have good reliability and validity were selected and used to collect the survey data. Specifically, the scale of the shared vision was referred to as the measurement items that were proposed by Mustakallio, Autio, and Zahra [17] and modified to suit the social entrepreneurial team context. It included three items. The scale of communication-cooperation was drawn on the basis of the scale that was developed by Lester et al. [48] for teamwork and adapted according to the practice of the social entrepreneurial team. The modified scale included eight items. The content of the items that measured the trust levels among social entrepreneurial team members adopted the items that were proposed by Carson et al. [49], including eight items. In this study, the participants were asked to evaluate the situation of their team’s relational governance in the past.

The content of the items to measure team learning was proposed by Kostopoulos et al. [50]. The scale includes 12 items, divided into 4 dimensions. Items 1 to 3 measure the intuition of the entrepreneurial team, Items 4 to 6 measure the interpretation level of the team, Items 7 to 9 focus on the integration level, and Items 10 to 12 test the coding level of the team. In this study, the scale has been adjusted to suit the entrepreneurial team learning status. In the pre-survey stage, the outer loading of Items 10 to 12 was found lower than 0.7 and hence were removed.

Considering the context of social entrepreneurial team governance and an organization’s ability to respond to crises, the measure items were drawn on the scale of organizational resilience that was developed by Kantur and Say [51]. On the basis of the social entrepreneurship context, 8 items were selected. Items 1 to 3 focus on the stability of enterprises in coping with internal and external crises, Items 4 to 6 measure the agility of enterprises to deal with crises, and Items 7 to 9 measure the overall integrity of enterprises in response to crises. In the pre-survey stage, the outer loading of Items 3 to 4 was found lower than 0.7 and were removed. Finally, 7 items were left.

The company age and company size were selected as control variables in the study. First, the maturity of a firm is usually reflected by its age. According to Hambrick and Mason [52], not only do firms at different stages of development face different environmental conditions, goals, and capabilities, but their performance in responding to crises might be also limited by the organizational structure and resource advantages. Second, as suggested by Atuahene-Gima and Murray [53], the larger the enterprise, the more advantages they have in source acquisition and construction to deal with crises.

### 3.2. Survey Implementation

In this study, data were collected through a web-based survey method, which is considered an effective way to collect survey information at a low cost [54,55]. The questionnaire was created and published on Questionnaire Star. A small-scale pre-survey was carried out before distributing the actual survey. In this stage, pairing data of social entrepreneur–top managers were collected, and then within-group interrater reliability (Rwg) was leveraged to test whether the evaluation of the constructs by different social entrepreneurial team members is consistent or not [56]. A total of 38 social entrepreneurial teams were selected, 76 questionnaires were distributed, and a total of 60 valid questionnaires from 30 teams were collected. The results showed that the median values of Rwg in shared vision, communication-cooperation, team identify, team learning, and organizational resilience were 0.896, 0.992, 0.969, 0.991, and 0.985, respectively, which are greater than the threshold of 0.7 [57]. The results showed that the evaluation data of social entrepreneurial members have good within-group interrater reliability. It is acceptable to collect data from only one member of a social entrepreneurial team in the next stage.

This study identified the target participants from two aspects. First, we reached the members of the social entrepreneurial team through the WeChat groups that were related to social entrepreneurship, and then further confirmed with them to make sure they are founders, shareholders, and top managers of social enterprises. Second, MBA class groups and alumni groups were leveraged to source more social entrepreneurs. Furthermore, the questionnaire link was set to be accessed only once per user. The official questionnaire was distributed to target participants from 15 July 2021 to 30 December 2021. All the participants were informed that the survey was anonymous, will not ask any questions about personal privacy, and that the data would be used for academic research only. If the participant selected the position “neither founder nor top manager,” or if the company age was older than 8 years, then the questionnaire was invalid and was excluded. In the pre-survey stage, 177 questionnaires were distributed and 130 were valid. In the official distribution stage, 524 questionnaires were sent to the social entrepreneurs, top managers, and stakeholders of social enterprises, and 317 pieces were collected. A total of 462 questionnaires were obtained, 66 invalid questionnaires were excluded, and 396 valid questionnaires were finally collected.

### 3.3. Statistical Analysis

First, the common method biases of the questionnaire were checked using SPSS 22.0. Unlike ordinary employees, top managers are hard to reach because their schedules are busy, which makes it more difficult to collect data from them, let alone the whole team. Thus, the questionnaire survey data were usually collected from one key entrepreneurial team member instead of collecting from the whole team [16,58]. Consequently, in this study, all the items were answered by the same person on the premise that the respondent is the founder, top managers, or shareholders of the social entrepreneurial team. Next, Harman’s single factor detection was used to check the common method bias status of the collected data [59]. Factor analysis was conducted on all items of the questionnaire, including shared vision, communication-cooperation, trust, team learning, and organizational resilience. The load of the first principal component without rotation was 36.26%, which is lower than 40%, indicating that the collected data did not have serious common method biases [59], and the analysis results are reliable.

Second, PLS-SEM was used to construct the structural equation model as well as to test the proposed hypotheses. CB-SEM is based on covariance analysis and estimates the constructed model by conducting the maximum likelihood method, which is mainly used to verify the theory. PLS-SEM combines the advantages of principal component analysis and multiple regression analysis. It is based on variance analysis and analyzed the model by partial least squares, which is mainly used for developing theory. Furthermore, PLS-SEM has obvious advantages, including allowing data without normal distribution, and algorithms are appropriate for small sample sizes as well as for complex models [60]. This study aims to explore the mechanism of social entrepreneurial team relational governance on organizational resilience. The construction in this study and the relationship between constructions are still in the initial stages. Therefore, the advantages of PLS-SEM on prediction analysis and theory development are suitable for the design of this study. In this study, the model convergent validity test includes the Cronbach’s alpha, composite reliability (CR), average variance extracted (AVE), and discriminant validity which were applied to make sure that the latent variables are empirically distinct from each other, and only when the squared root of the AVE of each latent variable is greater than the correlation between itself and the other latent variable is it acceptable [61]. In the end, IPMA was used to gain more insight into the results of the PLS-SEM and to identify the factors that were the most critical to the target constructs but do not perform well [62].

## 4. Results

### 4.1. Demographic Analysis

The valid survey data was from 75 cities in China, amongst them, respondents from Chengdu (16.92%), Beijing (14.65%), Hangzhou (7.07%), Shanghai (6.82%), and Shenzhen (6.31%) contributed more than half of the total. These cities are with the most developed social entrepreneurship in China. Furthermore, as shown in Table A2 of the Appendix A, the percentage of males is more than female social entrepreneurs, accounting for 58.3%, which is in line with the current gender ratio of social entrepreneurship in China. The age of the participants ranged from 21 to 50 years and above, with 52.78% of the social entrepreneurs aged between 31 and 40 years; most of the social entrepreneurial team members (87.63%) have a diploma degree and above; and 44.19% of them are founders, whereas 43.94% of them are the top manager of the company, which meets the requirement of the target respondents of this study. In terms of the age of social enterprises, 24.86% of the enterprises have been established for less than 3 years, 36.47% of the enterprises have been established for 3–5 years, and 38.67% of the enterprises have been established for 5–8 years. The sample distribution of social enterprises at all ages is relatively average. Among these enterprises, 41.44% had 2–20 employees, 21.55% had 21–50 employees, 22.93.40% had 51–200 employees, and 14.09% had more than 200 employees. Thus, the samples that were investigated in this study meet the research needs and are highly representative.

### 4.2. Reliability and Validity Analyses

Table 1 presents the results of the reliability and validity analyses. All the Cronbach’s α values of the SV, CC, TR, TL, and OR were over 0.70, indicating that the questionnaire had high reliability [63]. The values of AVE were over 0.50, while the CR values were over 0.8 [61]. Table 2 lists the values of discriminant validity. The diagonal elements in the matrix are the square roots of the AVE, and the results show that the value of each construct’s corresponding row and column is higher than the others [61]. Therefore, convergence validity and discriminant validity had satisfactory validity for all the constructs [61,63].

### 4.3. Model Verification

The collinearity test was carried out to make sure that the structural model was satisfactory for OLS regression. The results showed that both the inner and outer VIF values were lower than 3, indicating that the structural model met the collinearity test requirement [63]. Second, R square (R^2^) is one of the main criteria that is used to measure the prediction ability of the model; the larger the R^2^, the stronger the explanatory power of the model. The R^2^ of team learning and organizational resilience were 0.610 and 0.475, respectively, indicating that the model has strong explanatory power [64]. Third, the results of the cross-validated redundancy test showed that the Stone–Geisser’s Q^2^ of the endogenous latent variables of team learning and organizational resilience were 0.346 and 0.269, which means that the model has good prediction correlation. Finally, the standardized root means square residual of the model is 0.050, far below the critical value of 0.08, presenting that the model fit is satisfied [65]. Thus, the structural model has high prediction correlation, good fitness, strong model explanatory power, and is without collinearity issues.

The direct and indirect path coefficients of the structural model were identified by the PLS-SEM algorithm. Specifically, criteria of the standardized path coefficient, confidence intervals, T-value, and *p*-value were used to explore the relationship between the latent variables. As shown in Table 2, the *p*-values of the path coefficients indicate the validity of the model hypotheses, showing that the standardized path coefficients of all the hypotheses except H1a and H1b were greater than 0 and meet the 0.05 level of significance. This result indicates that the eight hypotheses of the model were acceptable, whereas H1a and H1b were rejected. Specifically, shared vision (β = 0.061, *p* > 0.05), communication-cooperation (β = 0.116, *p* > 0.05), and trust (β = 0.149, *p* < 0.05) positively affect organizational resilience, but the *p*-values of shared vision and communication-cooperation were higher than 0.05. Thus, the hypothesis H1c was verified, whereas hypotheses H1a and H1b were rejected. The better the entrepreneurial team’s relationship governance, shared vision (β = 0.220, *p* < 0.001), communication-cooperation (β = 0.385, *p* < 0.001), and trust (β = 0.260, *p* < 0.001), the more likely the team members are to engage in team learning, thus verifying hypotheses H2a, H2b, and H2c. Team learning has a strong positive effect on organizational resilience (β = 0.413, *p* < 0.001), verifying hypothesis H3. In addition, entrepreneurial team relationship governance effects organizational resilience through team learning through three intermediary paths, namely, shared vision (β = 0.091, *p* < 0.001), communication-cooperation (β = 0.159, *p* < 0.001), and trust (β = 0.107, *p* < 0.01). Thus, H4a, H4b, and H4c were verified. In conclusion, trust has positive effects on organizational resilience, and team learning plays partial mediating effects between them. The direct effect of shared vision and communication-cooperation on organizational resilience was not significant, whereas team learning played a completely mediating effect between them.

**Table 2:** Standardized path co-efficient and mediation effect of structural equation model.

Hypotheses	Standardized Path Coefficient	Sample Mean	Standard Deviation STDEV	T Statistics	Confidence Intervals CI	*p*-Values
Standardized path coefficient						
H1a SV -> OR	0.061	0.063	0.065	0.943	[−0.045–0.171]	0.173
H1b CC -> OR	0.116	0.120	0.082	1.417	[−0.012–0.253]	0.078
H1c TR -> OR	0.149	0.149	0.073	2.032	[0.030–0.268]	*
H2a SV -> TL	0.220	0.219	0.063	3.498	[0.111–0.319]	***
H2b CC -> TL	0.385	0.385	0.070	5.48	[0.262–0.493]	***
H2c TR -> TL	0.260	0.263	0.061	4.267	[0.167–0.365]	***
H3 TL -> OR	0.413	0.407	0.069	5.953	[0.287–0.514]	***
Mediation effect						
H4a SV -> TL -> OR	0.091	0.088	0.027	3.416	[0.044–0.132]	***
H4b CC -> TL -> OR	0.159	0.156	0.035	4.574	[0.102–0.215]	***
H4c TR -> TL -> OR	0.107	0.108	0.036	2.997	[0.055–0.172]	***

Note: SV = shared vision; CC = communication-cooperation; TR = trust; TL = team learning; OR = organizational resilience. * = *p* < 0.05; *** = *p* < 0.001.

### 4.4. Importance–Performance Map Analysis

IPMA is used to extend the results reporting of path coefficient estimates by adding a dimension that considers the average values of the latent variable scores. Specifically, the IPMA contrasts the total effects, representing the predecessor constructs’ importance in shaping a certain target construct, with their average latent variable scores indicating their performance [61,66]. The goal is to identify predecessors that have relatively high importance for organizational resilience but also have a relatively low performance, to gain more insights as well as to provide more targeted suggestions for practice. The results of IPMA indicate the priority management targets which help enterprises improve their capabilities to deal with crises.

According to Ringle and Sarstedt [66], a positive outer weight of the predecessors is a necessary condition for IMPA. In this paper, the results showed that the outer weights of shared vision, communication-cooperation, trust, and team learning were positive to organizational resilience. As shown in Table 3, the most important construct to organizational resilience is team learning (0.409), followed by communication-cooperation (0.271), trust (0.263), and shared vision (0.125). The construct with the highest performance is communication-cooperation (78.753), followed by shared vision (78.336), team learning (75.734), and trust (75.417). Therefore, considering the importance of the constructs to organizational resilience and their performance, team learning is the key factor to promote organizational resilience, but its performance needs to be strengthened. Furthermore, as shown in Appendix A, Table A2, the top three important indicators were TL2 (0.051), TL6 (0.049), and TL1, TL4, and TL7 (0.048), whereas their performance was 77.736, 76.263, 76.894, 77.567, and 75.549, respectively, which means indicators of TL2 (“In this team, I offered new ideas and solutions to complicated problems”) have to keep the existing practice, whereas TL6 (“In our team, we managed to have a productive and creative dialogue for entrepreneurial issues”), TL1 (“I could combine and synthesize diverse data, information, and ideas in this team”), and TL7 (“In our team, we managed to agree upon the action and activities that we should perform”) need further improving actions.

A comparison of the importance of the predecessors to team learning shows that communication-cooperation is the most important factor (0.383) and has the highest performance (78.753), which means that its performance is satisfactory. However, by looking at the importance–performance of each indicator, we found that the indicator TR8 (“The members understood that each would adjust to changing circumstances, even if not bound to change by the formal agreements”) was the most important one (0.043) but with low performance (77.384), which shows that the performance of communication-cooperation can be lifted by improving indicator TR8. Therefore, to promote the emergence of team learning and further strengthen organizational resilience, social entrepreneurial teams have to first improve their members’ understanding of environmental adaptability, and then build productive and creative dialogue to manage the entrepreneurial issues, to improve the team members’ capability in information integration, and to manage to agree upon the action and activities.

## 5. Discussion

Informal institutions are believed to be positively associated with the abilities of firms to deal with uncertainties and reduce the negative impacts of reverse factors on firms [15]. However, previous studies have rarely revealed how informal institutions promote the ability of an organization to deal with crises. Taking social entrepreneurial teams as the research object, this study reveals the roles of how entrepreneurial teams relational governance promotes organizational resilience by improving the level of team knowledge through team learning. This study reveals that team learning is the key factor in promoting organizational resilience, whereas communication-cooperation in relational governance is the most important factor to stimulate team learning. On the basis of institutional theory and the characteristics of the social entrepreneurial team, this study suggests that entrepreneurial team relational governance includes three dimensions, namely, shared vision, communication-cooperation, and trust. This study explores their role in promoting the ability of social start-ups to deal with crises as well as unpacks the mediating roles of team learning. The findings showed that the roles of each dimension of entrepreneurial team relational governance on organizational resilience and team learning are different. Although some studies focused on entrepreneurial team relational governance [1,16] and raise specific implementation methods, studies revealing the black box of the process, especially in the field of social entrepreneurship, are lacking. Therefore, this study provides a new direction for improving entrepreneurial team governance and its effect on social start-ups’ organizational resilience.

The results show that trust plays a significant role on organizational resilience directly, whereas the other two dimensions do not. Thus, hypothesis H1a is verified, whereas H1b and H1c are not. First, entrepreneurial team members will be consistent when they trust one another, which enables the team to respond to crises quickly, so as to improve the ability of social start-ups in leveraging the opportunities emerging from a crisis or reducing their negative impacts. Second, the growth of social start-ups is a tortuous curve toward their missions. A shared vision of a social enterprise is usually considered social value creation; however, in the face of crisis, social start-ups may give priority to their survival and may lead to their coping strategies being more economic orientation, which may confuse the entrepreneurial team members and result in team conflicts, and ultimately weaken social start-ups’ capacities to respond to crises. Thus, the effect of shared vision on organizational resilience is not significant. Finally, communication takes time and may slow social start-ups’ response to sudden breaks and makes them miss the opportunities to deal with a crisis. A crisis usually leaves no time for the entrepreneurial team members to make a decision through communication, which causes communication-cooperation to not improve the social start-ups’ organizational resilience directly. Similar to the findings of Zhu, Zhou, and Zhang [16], trust among entrepreneurial team members is always positively associated with firms’ development, while communication-cooperation and shared vision only work at a certain level. They argued that lower levels cannot stimulate the team members’ strength fully while higher levels might lead to polarization and discourage independent thinking. Since the levels of the two dimension have not been separated in this paper, it is not surprising that the results of this paper find that shared vision and communication-cooperation play nonsignificant effect on organizational resilience.

Second, all the dimensions of entrepreneurial team relational governance are positively associated with team learning, and hypotheses H2a, H2b, and H2c are verified. This finding supports the viewpoint of Senge [20] and Nonaka, o Nonaka, Ikujiro, and Takeuchi [18], that team learning can be cultivated by improving team atmosphere. Through entrepreneurial team relational governance, team members will have clearer understanding of one another and their organization, which may encourage them to communicate and cooperate, and thus strengthen their connection to promote complex knowledge sharing among the team [67]. The result of IPMA also supports that communication-cooperation is the most important factor to promote team learning. Therefore, through relational governance, individual heterogeneous knowledge is integrated into team knowledge and thus improves team cognition, which is considered the foundation of opportunity identification. Furthermore, hypothesis H3 (i.e., entrepreneurial learning is positively associated with organizational resilience) is verified in the context of social entrepreneurship. The result of IPMA suggests that team learning is the key factor to stimulate organizational resilience. The finding is consistent with the results of previous studies [11,43], that the role of team learning on organizational resilience is by improving the level of team cognition, which provides initiative abilities.

Finally, the results showed that team learning plays mediating roles between entrepreneurial team relational governance and organizational resilience. Thus, hypotheses H4a, H4b, and H4c are verified. Combined with the results of H1a, H1b, and H1c, it can be seen that team learning plays a partial mediating role between trust and organizational resilience, whereas it plays complete mediating roles in shared vision and communication-cooperation to organizational resilience, respectively. The findings confirm that the cognitive role bridges the effects of relational governance on organizational resilience [12,18]. Entrepreneurial team relational governance promotes the emergence of team learning and may improve the level of team cognition, which is considered fertile soil for cultivating social organizational resilience.

## 6. Conclusions

Entrepreneurial team learning plays a significant role to improve social start-ups’ organizational resilience, and relational governance is an effective way to stimulate entrepreneurial team learning. The results showed that trust was positively associated with organizational resilience, whereas shared vision and communication-cooperation was not. Trust, shared vision, and communication-cooperation were positively associated with team learning, and team learning was positively associated with organizational resilience. The mediator role of team learning between entrepreneurial team relational governance and social start-ups’ organizational resilience was confirmed. Thus, in the context of social entrepreneurship, this study suggests that relational governance improves the team atmosphere to encourage more positive interactions among team members, and finally enables them to cope with crises in innovative ways and in a timely manner.

### 6.1. Implications

There are two main theoretical contributions that are included in this paper. First, this study strengthens the application of institutional theory in the field of social entrepreneurship, especially in research on the sustainable development of social start-ups. Specifically, from the perspective of informal governance of institutional theory and according to the two unique characteristics of social start-ups, namely, focusing on social value creation and uncertainty in its development. This paper proposes that entrepreneurial team relational governance can better explain the role how social start-ups deal with unexpected issues by stimulating and integrating the capacities of the team members, and thus constructed the framework of entrepreneurial team relational governance, team learning, and organizational resilience. Second, the results of this study enrich the research on the relationship of the entrepreneurial team to organizational resilience from the perspective of entrepreneurial team relational governance. Previous studies have rarely focused on the role of entrepreneurial team relational governance in organizational resilience, and only a few studies revealed the mediating effect between the two, especially in the field of social entrepreneurship. In detail, the results showed that entrepreneurial team relational governance does not always promote the ability of an organization to deal with crises directly. Only improving the trust among team members has a significant effect on organizational resilience, whereas a shared vision and communication-cooperation of the team does not. However, entrepreneurial team learning plays a key role in organizational resilience and relational governance is significantly associated with team learning, especially in the dimension of communication-cooperation. Thus, entrepreneurial team relational governance improves organizational resilience mainly through the mediating path of team learning. Furthermore, despite studies on trust, intimacy, and communication among team members, this study believes that a social entrepreneurial team is not only a simple social exchange relationship but also a partnership that pursues social impact in both economic and social missions. Therefore, on the basis of the viewpoints of Blatt [1] and Zhu, Zhou, and Zhang [16], this study explored the mechanism of entrepreneurial team relational governance on organizational resilience from the dimension of shared vision and communication-cooperation comprehensively, and further verified the role of each dimension on organizational resilience by survey data. The findings reveal the key path and factors of the entrepreneurial team on organizational resilience.

The findings of this study have significant practical guidance for entrepreneurial teams and social start-ups. It can not only help the entrepreneurial team improve their knowledge to solve social problems innovatively but also enhances the ability of social start-ups to identify and leverage the opportunities that are brought by crises. For example, the COVID-19 pandemic has brought great challenges to the world and has resulted in a large number of social problems. On the one hand, these results showed that stimulating entrepreneurial team learning is a key factor to promote organizational resilience, and by comparing the scores of each indicator of the team learning scale, there are four most effective ways to stimulate entrepreneurial team learning that standout. They require that team members: (1) solve complicated problems with new ideas and solutions; (2) engage in a productive and creative dialogue to discuss issues; (3) integrate and synthesize diverse data, information, and ideas; and (4) agree upon the action and activities that should be performed. Thus, the entrepreneurial team must think innovatively, communicate effectively, welcome paradoxical knowledge, and respond quickly. On the other hand, communication-cooperation is the most important factor to stimulate entrepreneurial team learning; thus, team members should realize that each one of them must adjust to changing circumstances.

### 6.2. Limitations and Future Research Directions

As with any empirical study, this work has limitations. First, although we collected the data from multiple channels to widen the coverage of the data, considering that social entrepreneurship has only emerged in China and the regional distribution of existing social entrepreneurial teams is scattered, the data still failed to cover all regions, which may limit the generalization of the findings. Besides, this work was based on self-reported data, which may constrain the reliability of the results. Future research may consider in-depth interviews or longitudinal data for analysis to illustrate the dynamic process of social entrepreneurial team governance. Second, our work was based on a management context which may not fully illustrate the whole picture of social entrepreneurial team governance. Future work may conduct interdisciplinary research to explore more factors, such as economic and social factors that could promote or limit the results of social entrepreneurial team governance and its impact on organizational resilience in social entrepreneurial processes, to enrich the theory of entrepreneurial team relational governance, and provide more practical guidance to social enterprises.

## Figures and Tables

**Table 1:** Cronbach’s Alpha, convergence validity, composite reliability, and discriminant validity for the measurement model.

Construct	Number of Items	Cronbach’s Alpha	Convergence Validity AVE	Composite Reliability CR	Discriminant Validity				
					SV	CC	TI	TL	OR
SV	6	0.880	0.772	0.910	0.878				
CC	8	0.852	0.614	0.927	0.708	0.783			
TR	8	0.887	0.538	0.903	0.668	0.748	0.733		
TL	9	0.910	0.581	0.926	0.666	0.735	0.694	0.763	
OR	7	0.880	0.583	0.907	0.518	0.575	0.565	0.654	0.764

Note: the items on the diagonal represent the square roots of the average of variance extracted (AVE); off-diagonal elements are the Pearson correlation estimates. SV = shared vision; CC = communication-cooperation; TR = trust; TL = team learning; OR = organizational resilience.

**Table 3:** Importance and performance of each antecedent variable.

	Target Construct	Team Learning		Organizational Resilience	
Antecedent Variable		Importance	Performance	Importance	Performance
Shared vision		0.182	78.336	0.125	78.336
Communication-cooperation		0.383	78.753	0.271	78.753
Trust		0.269	75.417	0.263	75.417
Team learning		-	-	0.409	75.734

## Appendix A

**Table A1:** Scale content.

Construct	Item	Content
Shared vision (SV)	SV1	Team members share the same vision about the firm
	SV2	Team members are committed to jointly agreed-on goals of the firm
	SV3	Team members agree on the long-term development objectives of the firm
Communication -cooperation (CC)	CC1	Team members are very willing to share information about our work
	CC2	Team members are comfortable talking to each other about what needs to be done
	CC3	Team members enjoy talking to each other
	CC4	Team members cooperate to get the work done
	CC5	Team members work together to solve problems and make decisions
	CC6	Team members find it easy to work with each other
	CC7	When team members talk to each other, there is a great deal of understanding
	CC8	There is a lot of cooperation among members of my team
Trust (TR)	TR1	The members held mutual expectations about their responsibilities that went beyond what was specified in the formal agreements
	TR2	The members expected that conflicts would be resolved fairly, even if no guidelines were given by the formal agreements
	TR3	There were performance goals for the member’s work that were understood and accepted by the members even though not written in the formal agreements
	TR4	When an unexpected situation arose, the members had a mutual understanding that a win-win solution would be found, even if it contradicted the formal agreements
	TR5	The members were expected to share helpful information to an extent beyond that required by the formal agreements
	TR6	The members held mutual expectations that each would be flexible and responsive to requests by the other, even if not obliged by the formal agreements
	TR7	The members understood that problems arising during the relationship would be solved jointly through communication and cooperation rather than just reference to the formal agreements
	TR8	The members understood that each would adjust to changing circumstances, even if not bound to change by the formal agreements
Team learning (TL)	TL1	I could combine and synthesize diverse data, information, and ideas in this team
	TL2	In this team, I offered new ideas and solutions to complicated problems
	TL3	I could effectively improvise in this team
	TL4	In our team, we had a very good level of information and knowledge sharing
	TL5	In our team, we managed to develop a common picture/idea of the most critical problem and how they could be resolved
	TL6	In our team, we managed to have a productive and creative dialogue for entrepreneurial issues
	TL7	In our team, we managed to agree on the action and activities that we should perform
	TL8	In our team, we shared information, ideas, and results while performing entrepreneurial tasks
	TL9	During the project, our team developed and experimented with a number of different implementation scenarios, project deliverables, prototypes production designs
Organizational resilience (OR)	OR1	My organization stands straight and preserves its position
	OR2	My organization is successful in generating diverse solutions
	OR5	My organization is agile in taking required action when needed
	OR6	My organization is a place where all the employees engaged to do what is required from them
	OR7	My organization is successful in acting as a whole with all of its employees
	OR8	My organization shows resistance to the end in order not to lose
	OR9	My organization does not give up and continues its path

Note: SV = shared vision; CC = communication-cooperation; TR = trust; TL = team learning; OR = organizational resilience.

**Table A2:** Importance and Performance of each Indicator.

	Target Construct	Team Learning		Organizational Resilience	
Indicator		Importance	Performance	Importance	Performance
SV1		0.039	77.315	0.057	77.315
SV2		0.041	77.020	0.059	77.020
SV3		0.045	80.387	0.066	80.387
CC1		0.033	78.493	0.047	78.493
CC2		0.034	79.924	0.049	79.924
CC3		0.033	78.493	0.047	78.493
CC4		0.030	79.503	0.042	79.503
CC5		0.035	78.451	0.050	78.451
CC6		0.029	74.958	0.040	74.958
CC7		0.038	78.283	0.053	78.283
CC8		0.039	81.103	0.055	81.103
TR1		0.035	78.620	0.035	78.620
TR2		0.029	72.980	0.030	72.980
TR3		0.035	75.126	0.035	75.126
TR4		0.029	74.285	0.029	74.285
TR5		0.027	71.970	0.028	71.970
TR6		0.031	73.822	0.032	73.822
TR7		0.036	77.231	0.036	77.231
TR8		0.042	77.384	0.043	77.384
TL1		0.048	76.894	-	-
TL2		0.051	77.736	-	-
TL3		0.037	70.875	-	-
TL4		0.048	77.567	-	-
TL5		0.042	74.158	-	-
TL6		0.049	76.263	-	-
TL7		0.048	75.547	-	-
TL8		0.044	77.062	-	-
TL9		0.043	73.990	-	-

Note: SV = shared vision; CC = communication-cooperation; TR = trust; TL = team learning; OR = organizational resilience.
